# The Deleterious Influence of Tenofovir-Based Therapies on the Progression of Atherosclerosis in HIV-Infected Patients

**DOI:** 10.1155/2012/372305

**Published:** 2012-05-07

**Authors:** Gerard Aragonès, Pedro Pardo-Reche, Laura Fernández-Sender, Anna Rull, Raúl Beltrán-Debón, Esther Rodríguez-Gallego, Jordi Camps, Jorge Joven, Carlos Alonso-Villaverde

**Affiliations:** ^1^Unitat de Recerca Biomèdica (URB-CRB), Hospital Universitari Sant Joan, Institut Investigació Sanitària Pere Virgili (IISPV), Universitat Rovira i Virgili, 43201 Reus, Spain; ^2^Servei de Medicina Interna, Hospital Universitari Sant Joan, Institut Investigació Sanitària Pere Virgili (IISPV), Universitat Rovira i Virgili, 43204 Reus, Spain

## Abstract

We investigated the potential differential effects of antiretroviral therapies on unbalanced chemokine homeostasis and on the progression of atherosclerosis in HIV-infected patients. A two-year prospective study was performed in 67 consecutive HIV-infected patients initiating antiretroviral therapy with abacavir/lamivudine or tenofovir/emtricitabine. Circulating levels of inflammatory biomarkers, progression of subclinical atherosclerosis and expression levels of selected chemokines genes in circulating leukocytes were assessed. Control subjects showed significantly lower plasma concentrations of CRP, tPA, IL-6, and MCP-1 than HIV-infected patients at a baseline. After two years of followup, the observed decreases in plasma inflammatory biomarker levels were only significant for MCP-1, tPA, and IL-6. The decrease in plasma MCP-1 concentration was associated with the progression of atherosclerosis, and this effect was negligible only in patients receiving TDF-based therapy. Multivariate analysis confirmed that treatment with TDF was positively and significantly associated with a higher likelihood of subclinical atherosclerosis progression. However, the expression levels of selected genes in blood cells only showed associations with the viral load and total and HDL-cholesterol levels. Current antiretroviral treatments may partially attenuate the influence of HIV infection on certain inflammatory pathways, though patients receiving TDF therapy must be carefully monitored with respect to the presence and/or progression of atherosclerosis.

## 1. Introduction

Coronary artery disease (CAD) and the accumulation of classical cardiovascular risk factors are becoming increasingly common in human immunodeficiency virus (HIV)-infected patients [1]. However, these cardiovascular risk factors alone do not explain the increased incidence of CAD in these patients; classical risk scores typically underestimate the risk of coronary events in these patients, and there is a significant nonconcordance with subclinical atherosclerosis in HIV-infected subjects [2, 3]. Antiretroviral treatment-induced dyslipidemia has previously been considered sufficient to induce atherosclerosis in these patients; however, recent evidence suggests that both inflammation and specific responses to some antiretroviral treatment regimens play important roles in the development of the disease [4]. In particular, the identification of inflammatory biomarkers and the assessment of direct HIV-related damage to the arterial wall are currently subjects of intense research [5–7].

Clinical studies show that HIV infection, HIV treatment regimens and inflammation are independent factors associated with the development of atherosclerotic lesions [8]. It is also well documented that plasma concentrations of cytokines, including interleukin (IL)-6, tumor necrosis factor alpha (TNF-*α*), monocyte chemotactic protein 1 (MCP-1/CCL2), and IL-18, are higher in HIV-infected patients than in uninfected individuals; furthermore, these differences persist even in patients with well-controlled viral disease [9–12]. Considering both inflammation and lipid-related risk factors, we previously assessed the relationship between plasma concentrations of high-density lipoproteins (HDL) and HIV viral load [13]. Our results indicated that HDL particles modulate not only reverse cholesterol transport but also the inflammatory response [14, 15]. Moreover, at least in animal models, the anti-inflammatory action of HDL in the development of atherosclerosis seems to be more relevant than the actual enrichment of cholesterol [16]. The potential differential effects of antiretroviral therapies on unbalanced chemokine homeostasis and on the progression of atherosclerosis have not yet been investigated.

## 2. Materials and Methods

### 2.1. Study Participants

 We recruited patients from among the participants of an observational nonrandomized longitudinal project assessing atherosclerosis in HIV-infected patients [17]; all subjects agreed to participate in the present study and provided informed consent. The initial time-point of this study was the commencement of therapy including either abacavir lamivudine (ABC-3TC) or tenofovir emtricitabine (TDF-FTC). Candidates for inclusion were antiretroviral naïve patients, and patients previously exposed to antiretroviral treatment who had discontinued treatment for at least 6 months. No patient was being treated with lipid-lowering drugs at the commencement of the study, but 5 patients were treated with fluvastatin (80 mg/day) during the followup in ABC-3TC group and 3 in TDF-FTC group. For the purpose of this study, only patients who remained on the same antiretroviral treatment regimen during followup were included in the analysis. The study was approved by the Ethics Committee of the Hospital Universitari Sant Joan.

After informed consent forms were read and signed by the patients, complete physical examinations were performed, a fasting venous blood sample was drawn for analyses, and baseline scans of the carotid arteries were obtained. Data relating to smoking habits, alcohol consumption, opportunistic infections, hepatitis C virus (HCV) coinfection, the period of time since the original diagnosis, and specific therapies received by patients were collected. Laboratory analyses included lipid profiles, blood glucose concentration evaluations, assessments of inflammatory marker expression, plasma HIV-1 RNA level analysis, and blood CD4^+^ T cell counts. Serum HDL-cholesterol concentration was measured using a homogeneous assay based on the synthetic polymer/detergent method, version 3 (Beckman-Coulter, Fullerton, CA, USA), and LDL-cholesterol concentration was calculated using the Friedewald formula. Inflammatory marker levels and lipid profiles were compared with those obtained from sex and age-matched unrelated and uninfected control subjects who were recruited as described previously [18]. After two-year followup, a second ultrasonographic examination of the carotid arteries was performed. In addition, with the rationale that whole blood could reflect molecular changes that take place in response to different antiretroviral therapy in other key organs/tissues, a blood sample was obtained from each patient to evaluate the *CCL2*, *CCR5*, *CCR2,* and *CXCR-4* gene expression profiles of circulating leukocytes and to assess treatment-related differences as described below.

### 2.2. Measurement of Inflammatory Biomarkers

 EDTA-plasma was used for all of the measurements described. The concentration of C-reactive protein (CRP) was measured using a particle-enhanced turbidimetric immunoassay (Quantex hs-CRP kit, Biokit, Barcelona, Spain) with a sensitivity of 0.10 mg/L. Soluble CD40 ligand (sCD40L), IL-6, IL-8, MCP-1, soluble platelet selectin (P-selectin), and tissue plasminogen activator (t-PA) levels were measured using a Human Cardiovascular FlowCytomix Multiplex kit (Bender MedSystems, Vienna, Austria).

### 2.3. Standardized Ultrasound Protocol

 The carotid ultrasound measurement was performed essentially as described by Polak et al. [19] with the addition of some previously published modifications [20]. We used a GE Logiq 700 Ultrasound Machine with a 7- to 10-MHz ultrasound probe. We identified and digitally recorded the far wall of the common carotid artery (1 cm proximal to the bifurcation), the carotid bulb (in the bifurcation), and the internal carotid artery (1 cm distal to the bifurcation). All of the intimamedia thickness (IMT) measurements were performed at predefined points using AnaliSYS image processing software (Soft Imaging System, Münster, Germany). IMT measurements on each arterial segment were averaged and used in the statistical analyses as a combined IMT. To assess reproducibility of measurements, the images of 20 randomly selected patients were remeasured applying the same protocol. The intraclass correlation coefficient between the 2 sets of measurements was 0.91, and the absolute difference in IMT was 0.007 mm (0.018).

### 2.4. Gene Expression Analysis

 Three millilitres of blood were collected from subjects and directly deposited into TEMPUS blood RNA tubes (Applied Biosystems, Foster City, CA, USA). Total RNA was then isolated from these samples using an ABI PRISM 6100 Nucleic Acid PrepStation (Applied Biosystems). All TaqMan primers and probes were obtained from Assays-on-Demand (Applied Biosystems) and were used in a Micro Fluidic Card on a 7900HT Real-Time PCR system (Applied Biosystems). Micro Fluidic Cards were analysed with RQ documents and the RQ Manager Software. The expression levels of the *CCL2*, *CCR5*, *CCR2,* and *CXCR-4* genes were normalized to the expression of a designated endogenous control gene (*GAPDH*). Gene expression levels were calculated using the comparative threshold cycle (Ct) method.

### 2.5. Statistical Analysis

Analysis was performed using SPSS, version 19.0 (SPSS Inc., Chicago, IL). All data are presented as mean ± SD, except where otherwise stated. The Kolmogorov-Smirnov test was used to check whether data were normally distributed. Differences in baseline characteristics between the two treatment groups were assessed through the **χ*^2^* and Mann-Whitney *U* test for categorical and continuous variables, respectively. To asses whether changes in biomarkers levels were significantly different from baseline in each treatment group, and whether those changes were significantly different between both treatment groups, we used linear mixed models including the interaction between treatment group and study period as categorical. Univariate and multivariate analyses were performed, with adjustment for confounding factors such as sex, age, BMI, third antiretroviral drug, coinfection with HCV and lipid-lowering therapy. We calculated the difference in the combined IMT scores between the two examinations (delta IMT). For the assessment of factors that affect changes in IMT, the patients were divided into two groups, the *progressors* and *nonprogressors*, based on the mean annual increment of IMT (delta IMT, mm/year). *Progressors* were defined as those with a mean annual delta IMT ≥0.015 mm/year, whereas *non-progressors* as those with a delta IMT <0.015 mm/year according to the result of the meta-analysis which estimated the mean annual change in IMT among control groups from 13 published randomized placebo-controlled studies [21]. Patients were also grouped according to tertiles for HDL-cholesterol, total cholesterol, LDL-cholesterol, and triglycerides. Stepwise regression analysis was performed to identify prognostic factors for the delta IMT score. In the stepwise analysis method, variables were introduced one-by-one into the model; only variables that were found to be statistically significant remained in the model.

## 3. Results

### 3.1. General Characteristics of HIV-Infected Patients

 Of the 67 Caucasian subjects who met selection criteria, 35 (52.2%) initiated TDF-FTC and 32 (47.8%) ABC-3TC. The third component of the regimen was an nonnucleoside reverse transcriptase inhibitor (NNRTI) in 77% and 64% and a protease inhibitor (PI) in 33% and 46% patients treated with TDF-FTC and ABC-3TC, respectively. Thirty-eight patients (56.7%) were antiretroviral treatment naïve patients and 29 (43.3%) were patients reinitiating antiretroviral therapy after treatment interruption for at least 6 months. Of the 67 patients initially selected, data obtained from 13 patients were excluded due to cardiovascular disease events (*n* = 2), unstable followups (*n* = 8) or poor quality of the recorded images (*n* = 3). No significant differences were observed between antiretroviral treatment groups for the variables considered here. The main clinically relevant characteristics of the patients included in the study are summarized in Table 1. The mean time from diagnosis was 7.24 ± 0.36 years, and 38 (56.7%) patients were coinfected with HCV. These patients were either current or past intravenous drug users (59%) or became infected as a result of sexual intercourse.

The baseline examination revealed that most patients were heavy smokers, were relatively young, were not significantly obese, and had normal blood pressure values. The mean plasma lipid and glucose concentrations were determined to be within laboratory reference ranges. Demographic-, HIV-, or cardiovascular risk-related baseline characteristics were not significantly different between patients on TDF-FTC- or ABC-3TC-based regimens (Table 1). However, control subjects showed significantly lower plasma concentrations of CRP (*P* < 0.001), tPA (*P* < 0.001), IL-6 (*P* = 0.04), and MCP-1 (*P* = 0.03) than HIV-infected patients at the baseline time point (Table 1).

### 3.2. Variables Related to the Progression of Atherosclerosis

There was a significant mean annual increase (0.045 mm) in the IMT between the baseline measurements [0.77 (0.01) mm] and the followup values [0.85 (0.01)]. Baseline values were not significantly influenced by the inflammatory markers or the lipid profile. We used the delta IMT to segregate the patients into *progressors* [delta IMT = 0.22 (0.01) mm] and *nonprogressors* [delta IMT = −0.05 (0.02) mm] so as to identify variables that may influence the course of atherosclerosis (Table 2). None of the classical cardiovascular disease risk factors were significantly associated with the course of IMT. When patients were stratified by circulating levels of total, LDL-, and HDL-cholesterol, the proportion of patients with high total and LDL-cholesterol concentrations was higher among the *progressors* in both groups of patients, but the differences did not reach statistical significance. Only the use of TDF was significantly associated with the progression of subclinical atherosclerosis (Table 2). The TDF group showed a higher proportion of *progressors* (61%) with respect to ABC group (33%; *P* = 0.003). When we applied multivariate stepwise regression analysis to identify variables that could be associated with the progression of atherosclerosis in HIV-infected patients, the results confirmed that TDF-based treatment was positively and significantly associated with a higher likelihood of subclinical atherosclerosis progression (OR = 7.1, *P* = 0.002, 95% CI: 2–24).

### 3.3. Variables Related to Inflammatory Biomarkers

**Figure 1:**
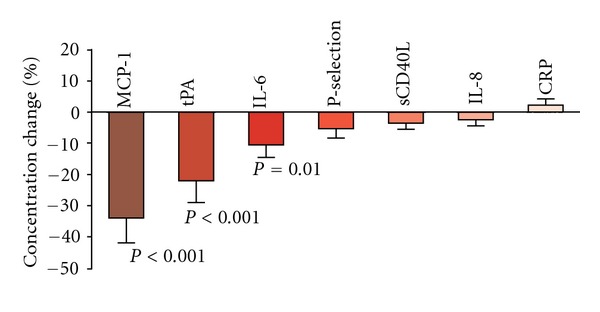
Changes in plasma concentrations of inflammatory markers after two years of followup. The observed decreases in plasma concentrations of inflammatory biomarkers (expressed as percentages) were only significant for MCP-1, tPA, and IL-6.

**Figure 2:**
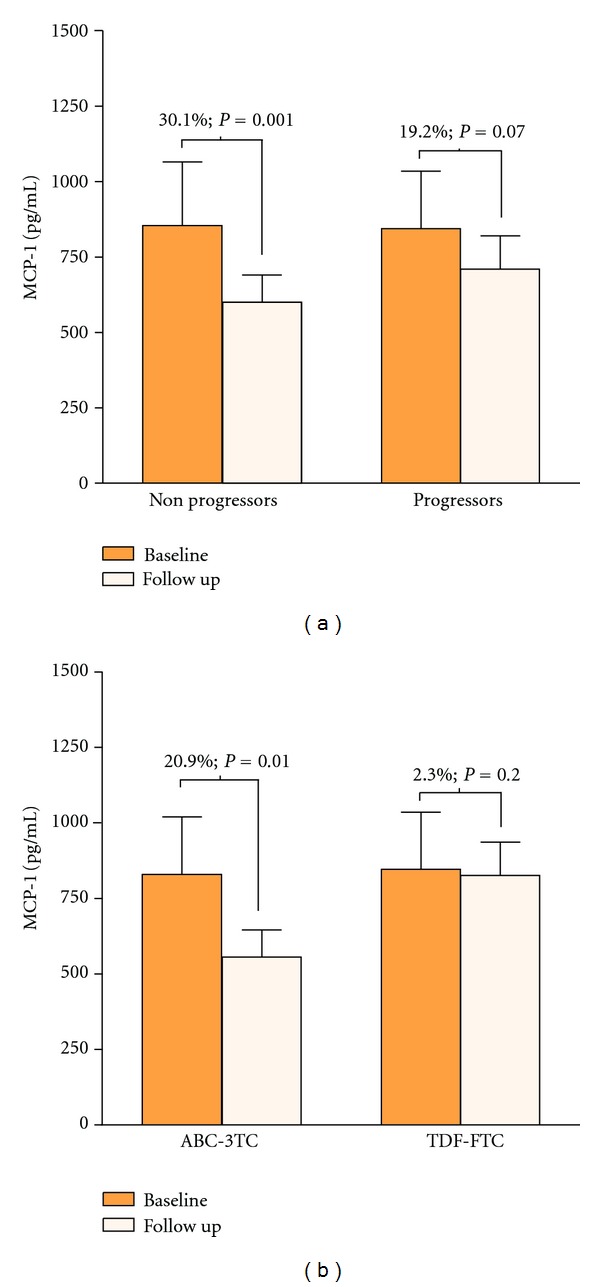
Changes in plasma concentration of MCP-1 after two years of followup in HIV-infected patients. A decrease in plasma MCP-1 concentration was observed in all HIV-infected patients, though it was more prominent and significant in those who did not display progression of atherosclerosis (a). Stratification according to antiretroviral treatment strategy (b) revealed that the decrease in plasma MCP-1 concentration was not significant in patients receiving TDF-FTC.

In HIV-infected patients, but not in control subjects, the plasma concentration of HDL-cholesterol was found to be negatively correlated with the expression levels of tPA (*ρ* = −0.3, *P* = 0.001), MCP-1 (*ρ* = −0.25; *P* = 0.002), P-selectin (*ρ* = 0.17, *P* = 0.018), IL-6 (*ρ* = −0.18, *P* = 0.016), and IL-8 (*ρ* = −0.17, *P* = 0.013). No correlation was found with other lipid-related parameters, and no significant differences in the concentrations of inflammatory biomarkers related to the antiretroviral treatment group were observed at baseline. In the second evaluation, the mean CD4^+^ T-cell count was significantly increased [462.9 (27.1) versus 523.6 (27.8) in cell/*μ*L, *P* < 0.001], 89% of patients exhibited a HIV viral load <200 copies/mL, and total plasma and LDL-cholesterol levels were found to be significantly decreased in both groups of antiretroviral treatment. Interestingly, after two years of followup, the observed decreases in the plasma concentrations of inflammatory biomarkers were only significant for MCP-1 (*P* < 0.001), tPA (*P* < 0.001) and IL-6 (*P* = 0.01) (Figure 1). Accordingly, although plasma MCP-1 levels were decreased in all patients (Figure 2(a)), this decrease was not significant among *progressors* [845.6 (205.3) pg/mL at baseline *versus* 710.9 (115.6) pg/mL at followup; *P* = 0.079]. Furthermore, as shown in Figure 2(b), the decrease in MCP-1 levels were significantly smaller in patients initiating TDF-FTC [846.4 (198.1) pg/mL at baseline *versus* 826.3 (113.9) pg/mL at followup; *P* = 0.2] compared with those starting ABC-3TC [830.8 (193.3) pg/mL at baseline *versus* 555.1 (96.2) pg/mL at followup; *P* = 0.01].

### 3.4. Gene Expression Analysis in Circulating Leukocytes

**Figure 3:**
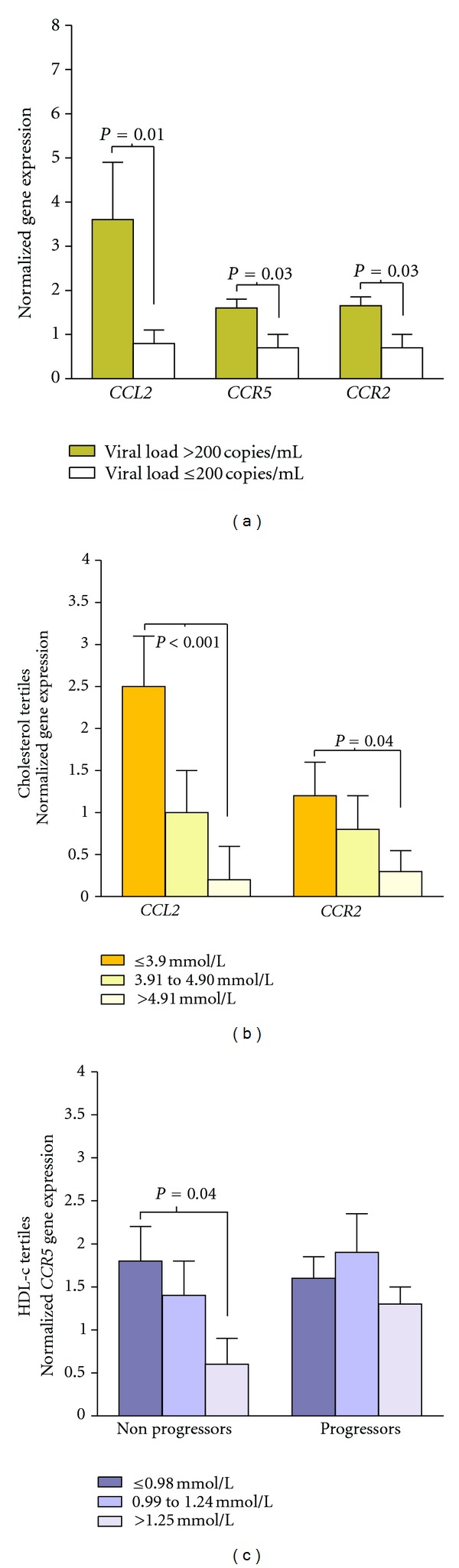
Changes in the expression levels of *CCL2*, *CCR2,* and *CCR5* genes. The expression levels of selected cytokine-related genes in blood cells of HIV-infected patients in relation to HIV viral load (a), total cholesterol levels (b), and HDL-cholesterol (HDL-c) concentration (c).

To test if these results could be explained by changes in blood cell gene expression patterns, we measured *CCL2*, *CCR5*, *CCR2,* and *CXCR-4* gene expression levels. We found no differences between the antiretroviral treatment groups. HIV-related characteristics, such as HCV coinfection and the time and route of infection, were also unrelated to the gene expression patterns. However, we observed significantly lower *CCL2*, *CCR5*, and *CCR2* gene expression levels in patients exhibiting measurably better virological control (Figure 3(a)). Surprisingly, we also found that *CCL2* and *CCR2* gene expression levels were lower in patients with the highest cholesterol levels (Figure 3(b)). In addition, lower *CCL2* gene expression levels were observed in patients with the highest plasma concentrations of LDL-cholesterol (*P* = 0.023) and plasma triglycerides (*P* = 0.04). In contrast, lipid parameters were not correlated with the expression levels of *CCR5* or *CXCR-4*. We further assessed the role of lipid variables in the level of *CCL2* gene expression, and we found a negative correlation with total cholesterol concentrations in patients receiving ABC-3TC (*ρ* = −0.5, *P* = 0.004). In contrast, plasma HDL-cholesterol concentrations were positively correlated with MCP-1 gene expression levels (*ρ* = 0.6, *P* = 0.006) in patients receiving TDF-FTC. Finally, *nonprogressors* with the highest plasma HDL-cholesterol concentrations presented a significant decrease in *CCR5* gene expression levels (*P* = 0.04) (Figure 3(c)).

## 4. Discussion

New therapies have dramatically decreased the mortality of patients infected with HIV, though recent findings suggest that several treatment-associated alterations may play a role in future morbidity and life expectancy. Among these alterations, the presence and/or progression of atherosclerosis with subsequent risk of myocardial infarction is a matter of concern [1–8]. Predisposing factors, such as lipoprotein disturbances and altered circulating cytokine profiles and monocytes subsets, have been previously demonstrated to occur in response to specific treatments and in response to HIV infection itself [9–12]. Our data indicate that, regardless of the treatments administered, effects are observed for some cytokines but not for others. Infected patients exhibited increased plasma concentrations of CRP, tPA, IL-6, and MCP-1, but other cytokines (i.e., P-selectin, sCD40L, IL-8) seemed to be unresponsive to HIV infection. The mechanisms that trigger the selective production of cytokines, and consequently influence the overall inflammatory response, remain undefined, though our findings suggest that these may be associated, at least partially, with the well-documented anti-inflammatory role of HDL [15]. In this sense, the patients involved in this study showed low HDL-cholesterol levels relative to controls, and significant correlations between plasma levels of HDL-cholesterol and most of the measured cytokines were observed, suggesting that HDL particles may lose their anti-inflammatory properties in HIV infection. During metabolic/inflammatory stress, HDL may become dysfunctional, exhibiting proinflammatory and consequently proatherogenic effects. Altered HDL cholesterol concentrations are common in HIV-infected patients and are usually attributed to the effects of both antiretroviral drugs and HIV infection itself [22, 23]. It is not fully understood whether HDL particle distribution may add relevant information to the function of HDL in the absence or presence of HIV treatment, and whether such effect adds further significance to the accelerated atherosclerosis observed in these patients. However, our results are concordant with previous findings that higher plasma MCP-1 concentration is associated with higher rates of atherosclerosis [20]. The lack of correlation with plasma concentrations of CRP and sCD40L observed in this study may be explained by the complementary disturbances in endothelial cell and liver functions that are frequently observed in HIV-infected patients [24–26].

In the present study, we also found that continuous antiretroviral therapy may substantially reduce the plasma concentrations of some cytokines, thus attenuating the levels of surrogate cardiovascular risk markers. The observed effect on plasma MCP-1, IL-6, and tPA concentrations was high, but the effect on other cytokines tested was negligible. After two years of followup, the decrease in plasma tPA and IL-6 concentrations was found to be unrelated to the treatment regimen given, and the effects on both were quantitatively moderate. However, plasma MCP-1 concentrations only decreased in those treated patients who did not receive TDF. This is concordant with experimental studies showing that TDF may increase the production of MCP-1 via an NF-*κ*B-related pathway [27]. Such effects, which we have confirmed clinically, should be considered deleterious. As shown by our results, the reduction in plasma MCP-1 levels is greater in those subjects considered to be *nonprogressors* in comparison to subjects in which atherosclerotic lesions progressed. Moreover, multivariate analysis confirmed that TDF treatment was positively and significantly associated with a higher likelihood of atherosclerosis progression. Such a relationship is plausible according to previous findings. The absence of MCP-1 or its receptor, CCR2, in susceptible mice protects against the development of atherosclerotic lesions, a condition in which macrophage recruitment and lipid overload play crucial roles. It has been also demonstrated that the MCP-1/CCR2 signalling pathway may represent a common pathway for many proatherogenic factors and that this pathway plays a central role in monocyte recruitment, lesion formation, and vascular repair [28, 29]. In HIV-infected patients, genetic variations in the *CCL2*/*CCR2* pathway have been associated with survival, and polymorphisms that increase the expression of *CCL2 *have been repeatedly associated with the presence of subclinical atherosclerosis [30–32].

When we assessed the expression levels of relevant genes in blood cells, we found that a detectable viral load is a determinant of the relative expression levels of the *CCL2*, *CCR2,* and *CCR5* genes. Furthermore, this modulation was not related to the treatment regimen group investigated in our study. This result is in accordance with the well-established finding that viral proteins such as the transactivator of transcription (Tat) protein may increase the *in vitro* expression of CCR5 and CXCR-4, the main coreceptors for HIV [33]. It is particularly noteworthy that CCR5 is an emerging therapeutic target; critically, blocking agents targeting this receptor may inhibit not only HIV replication but also atherosclerotic progression, a pleiotropic effect that should be thoroughly considered [34]. It is also of note that in our patients the relative decrease in the CCR5 gene expression levels was associated with higher HDL-cholesterol levels, an effect that was observed only among *nonprogressors*. There was also a concomitant association between decreased *CCL2* and *CCR2* gene expression levels and higher total cholesterol levels; this effect deserves further research and is likely related to the total burden of oxidized LDL as previously described [23].

Finally, it is important to note that changes that occur in monocytes and macrophages during HIV infection are also likely to impact on atherogenic processes [35], and we have not assessed them in the present study. The CD14^+^/CD16^+^ “proinflammatory” monocyte subpopulation is preferentially susceptible to HIV infection and may play a critical role in the pathogenesis of HIV-related cardiovascular disease. Moreover, both *CCR5* and *CCR2* expression levels correlate significantly with CD14^+^/CD16^+^ monocyte subset [36], and metabolic abnormalities such as dyslipidemia and insulin resistance, have been also reported to promote an inflammatory phenotype in circulating monocytes [37].

A limitation to this study is that the number of patients included was relatively low and eight of them were treated with statin during the followup. These aspects limit the validity of the conclusions, which should be confirmed in controlled larger populations. Another drawback was the nonrandomized assignment of therapy groups, which could have influenced the significant differences observed between groups.

In conclusion, TDF-based therapy had no beneficial effect on circulating MCP-1 concentration and was significantly associated with a higher likelihood of subclinical atherosclerosis. Our data reinforce the notion that HIV infection modulates factors that strongly influence certain inflammatory pathways [38–40] and reflect the complexity of phenomena accompanying antiretroviral therapy initiation in viremic patients, which might suggest a closer monitoring with respect to cardiovascular disease risk during the initial phases of treatment.

## Figures and Tables

**Table 1:** Characteristics of the control group (*n* = 151) and HIV-infected patients (*n* = 67) at baseline and at follow-up by treatment group.

		HIV-infected patients			
Characteristics	Control group	ABC-3TC (*n* = 35)		TDF-FTC (*n* = 32)	
		Baseline	Followup	Baseline	Followup
Gender, male (%)	135 (67)	24 (68)	NA	20 (62)	NA
Age, years	39.1 (0.4)	38.9 (0.6)	NA	38.3 (0.6)	NA
BMI, kg/m^2^	26.2 (0.2)	23.31 (0.2)	23.52 (0.4)	23.21 (0.2)	23.82 (0.3)
Systolic blood pressure, mm Hg	119.14 (1.7)	118.04 (1.4)	120.46 (1.5)	118.45 (1.5)	121.01 (1.5)
Diastolic blood pressure, mm Hg	77.36 (1.2)	75.96 (1.1)	78.35 (1.9)	76.26 (1.4)	78.01 (0.97)
Smoking, *n* (%)	90 (39.7)^a^	23 (66)	21 (62)	20 (62)	19 (59)
Total WBC count, cells x 10^9^/L	NA	4.27 (1.6)	4.73 (2.1)	4.50 (1.9)	4.83 (2.0)
CD4^+^ T cell count, cells/*μ*L	NA	458.97 (20.0)^b^	526.33 (24.8)	469.87 (20.2)^b^	520.25 (26.8)
Total cholesterol, mmol/L	5.02 (0.3)	5.06 (0.1)^b^	4.90 (0.1)	4.99 (0.1)^b^	4.80 (0.1)
HDL cholesterol, mmol/L	1.47 (0.1)^a^	1.17 (0.04)	1.19 (0.04)	1.18 (0.04)	1.19 (0.03)
LDL cholesterol, mmol/L	2.78 (0.3)	2.82 (0.1)^b^	2.68 (0.1)	2.88 (0.1)^b^	2.58 (0.1)
Triglycerides, mmol/L	1.1 (0.3)	2.50 (0.2)	2.53 (0.2)	2.49 (0.2)	2.59 (0.2)
Glucose, mmol/L	4.91 (0.4)	5.42 (0.1)	5.40 (0.2)	5.42 (0.2)	5.35 (0.1)
CRP, mg/L	0.83 (0.1)^a^	4.67 (3.4)	4.81 (3.9)	4.85 (3.2)	4.76 (4.2)
MCP-1, pg/mL	427.9 (149.7)^a^	830.8 (193.3)^b^	555.1 (96.2)	846.4 (198.1)	826.3 (113.9)
sCD40L, ng/mL	2.03 (0.2)	1.64 (0.4)	1.56 (0.3)	1.60 (0.5)	1.71 (0.7)
tPA, ng/mL	5.63 (0.5)^a^	9.71 (0.86)^b^	6.60 (0.64)	9.45 (0.79)^b^	6.10 (0.64)
IL-6, pg/mL	3.29 (0.8)^a^	7.19 (1.3)^b^	6.19 (1.3)	7.11 (1.5)^b^	6.30 (1.1)
IL-8, pg/mL	14.88 (6.1)	36.38 (5.2)	32.92 (8.1)	36.99 (7.3)	34.01 (7.9)
P-Selectin, pg/mL	185.23 (50.4)	205.19 (88.8)	199.05 (70.9)	211.11 (79.8)	200.35 (72.6)

Data are the mean (SEM) unless otherwise indicated. ^a^ *P* < 0.05 with respect to baseline values of all HIV-infected patients; ^b^ *P* < 0.05 with respect to follow-up values. BMI, body-mass index; NA, not applicable; WBC: white blood cells.

**Table 2:** Comparison of baseline values in groups segregated by the course of IMT.

Characteristics	Nonprogressors (*n* = 21)	Progressors (*n* = 46)
Gender, male (%)	14 (67)	30 (65)
Age, years	40.1 (1.1)	39.9 (1.1)
BMI, kg/m^2^	22.9 (0.2)	23.1 (0.2)
Systolic blood pressure, mm Hg	119.9 (2.7)	115.9 (1.9)
Diastolic blood pressure, mm Hg	79.6 (1.4)	77.3 (1.3)
Smoking, *n* (%)	13 (62)	30 (65)
TDF-FTC-based therapy, *n* (%)	7 (33)	28 (61)*
CD4^+^ T cell count, cells/*μ*L	463.2 (46.1)	469.9 (19.3)
Total cholesterol, mmol/L	4.79 (0.3)	5.02 (0.4)
HDL cholesterol, mmol/L	1.18 (0.2)	1.17 (0.1)
LDL cholesterol, mmol/L	2.66 (0.3)	2.79 (0.4)
Triglycerides, mmol/L	2.48 (0.3)	2.51 (0.3)
Glucose, mmol/L	5.40 (0.4)	5.41 (0.4)
Delta IMT, mm	−0.05 (0.01)	0.22 (0.01)*

Data are the mean (SEM) unless otherwise indicated. ^a^ *P* < 0.05 with respect to nonprogressors BMI, body-mass index.

## Abbreviations

3TC: Lamivudine
ABC: Abacavir
BMI: Body mass index
CAD: Coronary artery disease
CCL2: Chemokine CC motif ligand 2
CCR2: Chemokine CC motif receptor 2
CCR5: Chemokine CC motif receptor 5
CXCR-4: Chemokine CxC motif receptor 4
CRP: C-reactive protein
FTC: Emtricitabine
HCV: Hepatitis C virus
HDL: High-density lipoprotein
HIV: Human immunodeficiency virus
IL-6: Interleukin-6
IL-8: Interleukin-8
IMT: Intima media thickness
MCP-1: Monocyte chemotactic protein-1
sCD40L: Soluble CD40 antigen ligand
TDF: Tenofovir
tPA: Tissue plasminogen activator.
